# Usability Study of a Computer-Based Self-Management System for Older Adults with Chronic Diseases

**DOI:** 10.2196/resprot.2184

**Published:** 2012-11-08

**Authors:** Calvin Or, Da Tao

**Affiliations:** 1 Department of Industrial & Manufacturing Systems EngineeringThe University of Hong KongPokfulamChina (Hong Kong)

**Keywords:** Usability evaluation, self-management, patient participation, chronic disease

## Abstract

**Background:**

Usability can influence patients’ acceptance and adoption of a health information technology. However, little research has been conducted to study the usability of a self-management health care system, especially one geared toward elderly patients.

**Objective:**

This usability study evaluated a new computer-based self-management system interface for older adults with chronic diseases, using a paper prototype approach.

**Methods:**

Fifty older adults with different chronic diseases participated. Two usability evaluation methods were involved: (1) a heuristics evaluation and (2) end-user testing with a think-aloud testing method, audio recording, videotaping, and interviewing. A set of usability metrics was employed to determine the overall system usability, including task incompletion rate, task completion time, frequency of error, frequency of help, satisfaction, perceived usefulness, and perceived ease of use. Interviews were used to elicit participants’ comments on the system design. The quantitative data were analyzed using descriptive statistics and the qualitative data were analyzed for content.

**Results:**

The participants were able to perform the predesigned self-management tasks with the current system design and they expressed mostly positive responses about the perceived usability measures regarding the system interface. However, the heuristics evaluation, performance measures, and interviews revealed a number of usability problems related to system navigation, information search and interpretation, information presentation, and readability. Design recommendations for further system interface modifications were discussed.

**Conclusions:**

This study verified the usability of the self-management system developed for older adults with chronic diseases. Also, we demonstrated that our usability evaluation approach could be used to quickly and effectively identify usability problems in a health care information system at an early stage of the system development process using a paper prototype. Conducting a usability evaluation is an essential step in system development to ensure that the system features match the users’ true needs, expectations, and characteristics, and also to minimize the likelihood of the users committing user errors and having difficulties using the system.

## Introduction

With the advent of advanced technology, a number of health information systems have been developed and employed to increase support for patient self-management of chronic disease. However, many of those innovations are not regularly used in care management and some have been abandoned. This non-adoption issue is significant and can largely be attributed to problems with the usability of the technology, such as ineffective system design, lack of ease of use and convenience of access, and a mismatch between the system features and the needs, expectations, and characteristics of the users [1,2]. Even when a technology is adopted, these usability barriers are likely to result in frustration and irritation for the user, in inefficiency and disruption in the care management process, and in a higher likelihood of committing errors [3].

To avoid these negative outcomes, designers should evaluate and verify system usability during the early stages of system development [4]. This is especially important for health care technologies because their usability can have implications for quality and effectiveness of health care [5-7]. In fact, researchers have directed their efforts at improving the usability of their new health information technology (IT) applications to avoid unintended consequences at rollout [8-14]. For example, Tang and colleagues [12] applied the heuristics evaluation, a usability engineering method, to examine the usability of a digital emergency medical service system designed for paramedics to input patient data. They uncovered a number of heuristic violations in the user interface design. In another health care IT project, Rose and colleagues [11] conducted a qualitative study to assess the usability of a Web-based electronic medical record and used the findings to recommend design changes to the system. Similarly, Yen and Bakken [13] performed a heuristics evaluation and think-aloud test to study the usability of a Web-based communication system for nurse scheduling. They demonstrated that their study was effective in identifying system design problems and obstacles to task performance.

Usability is also important for elderly and disabled people for the following reasons. First, most older adults and others with disabilities are experiencing a decline in their physical and cognitive abilities [15,16]; as a result, they may have more difficulty interacting with technology [17,18]. Second, many technologies are not made to be accessible for these people, making it difficult to use them [18,19]. Third, many of the design guidelines are established for developing products for people with no functional limitations; thus, it is necessary to pay special attention to the usability of the products that are specifically designed for the elderly and disabled. Indeed, a number of researchers who are interested in aging, disability, and technology demonstrate the effectiveness of usability evaluation in technology development [20-25].

Most of these previous works cover either Web sites or health care provider technology, but our study focuses on the usability evaluation of a patient-centered interactive self-management system for older adults with chronic illnesses. We focus on this because we acknowledge the high prevalence of chronic diseases among the elderly [26] and the potential for using health IT to improve disease self-management and health outcomes of elderly patients [27,28].

Usability evaluation includes a set of techniques for improving the usability of a system through the identification of potential difficulties and problems in using the system [4,29]. Among the various techniques, end-user testing and heuristics evaluation are prevalent and prominent [30,31]. End-user testing examines how effective and efficient a task or process is carried out using the system and explores users’ opinions based on their experience with the system. Heuristics evaluation is performed by usability specialists and focuses on the assessment of the system against a set of human factors design guidelines and heuristics [4,32]. These two methods can be implemented together in a usability evaluation to increase the likelihood of uncovering more design problems [30,33].

Conducting a usability evaluation during the early stages of the development process for a new design is highly recommended [29]. In addition, using paper prototypes to study usability is practical due to their low cost and comparable effectiveness with computer-based prototypes in identifying usability problems [34-38]. This study, which was part of a larger project to develop a computer-based self-management system for older adults with chronic diseases, evaluated the usability problems and weaknesses of the system using a paper prototype test. We first conducted a heuristics evaluation and then end-user testing using the think-aloud method. The objective of the heuristics evaluation was to determine whether the system design characteristics met the human factors design guidelines and principles. The aim of end-user testing was to examine use performance and satisfaction with the system interface among a group of elderly patients with chronic diseases. This usability study analytically discovered design weaknesses in the self-management system and provided directions for system design modifications and for conducting future system analyses.

## Materials and Methods

### Self-management System Paper Prototype

**Figure 1:**
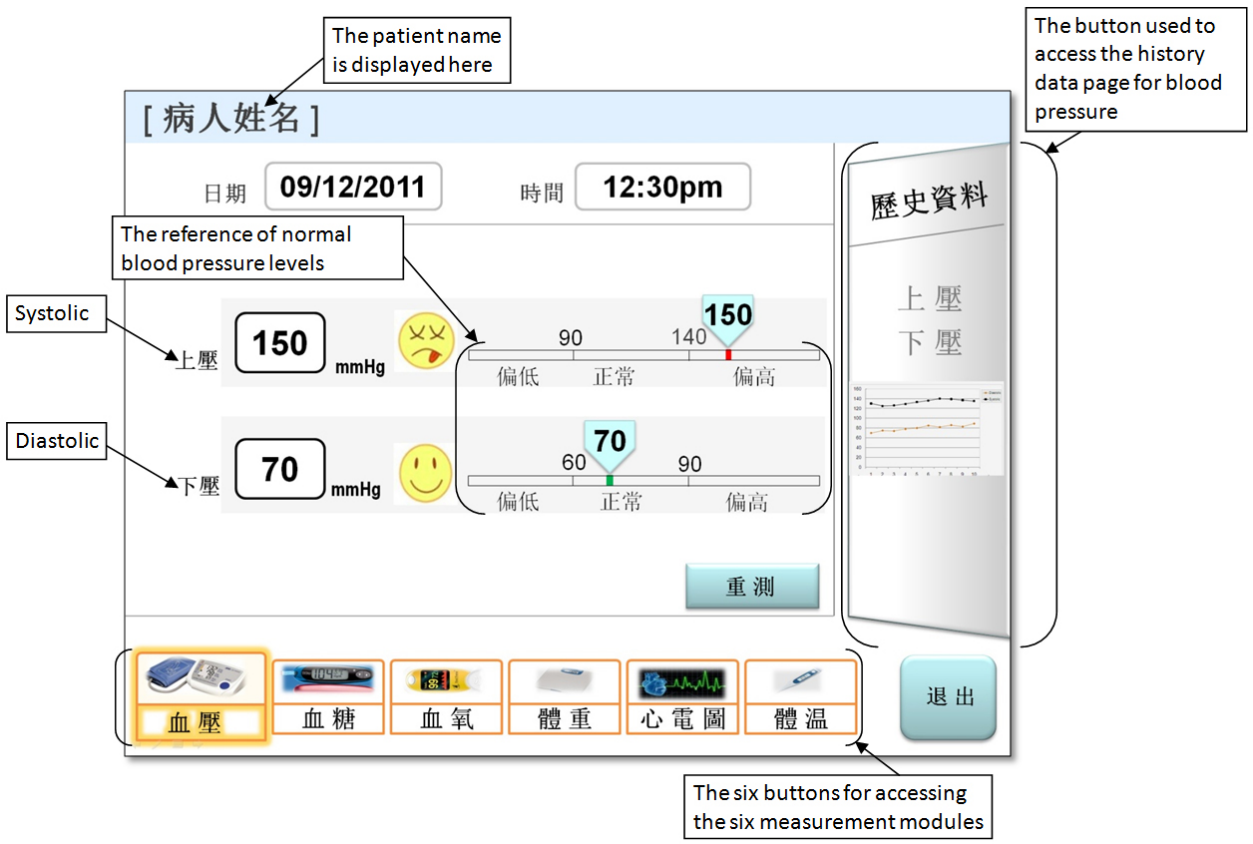
The blood pressure measurement page of the self-management system.

**Figure 2:**
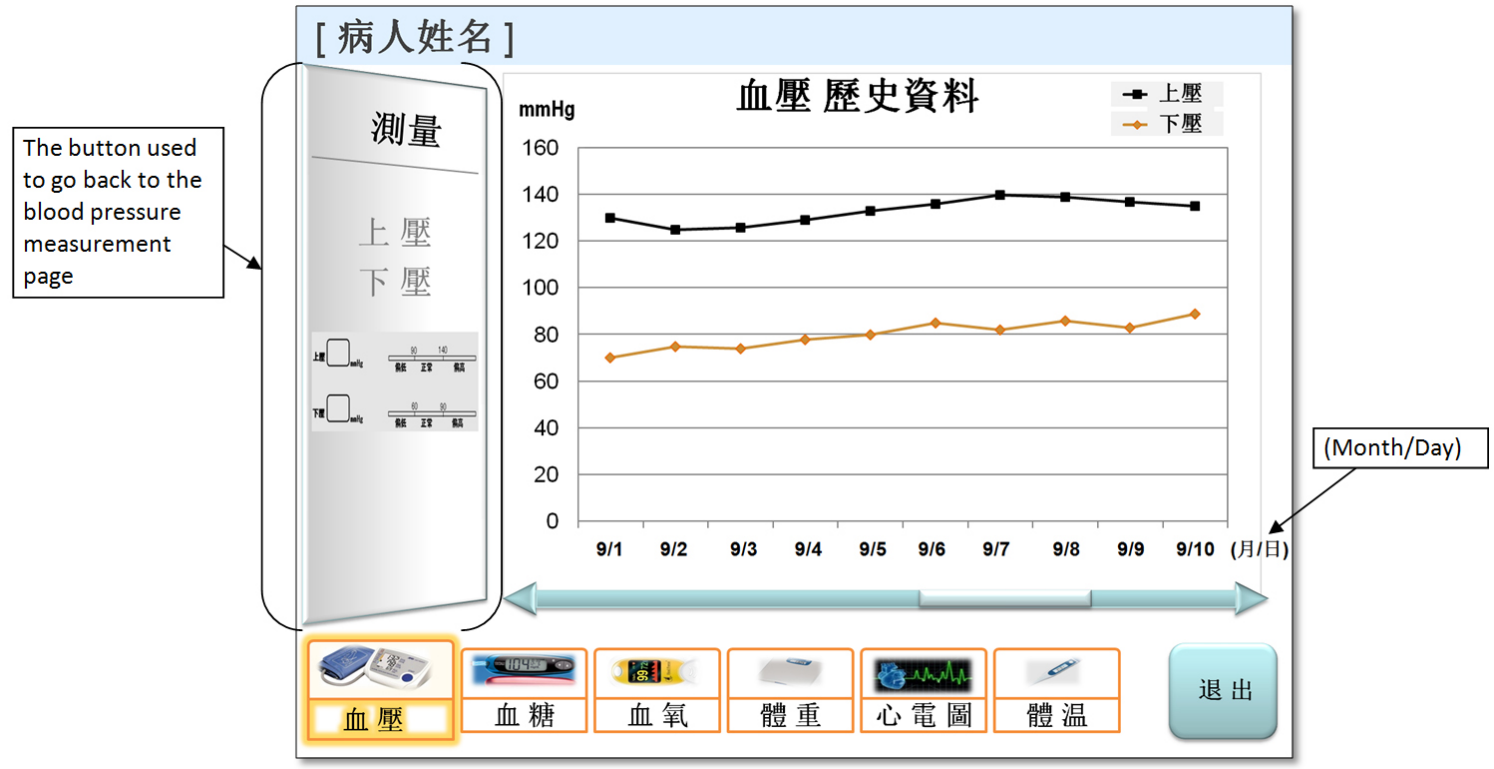
The blood pressure history data page presents the past blood pressure values on a two-dimensional line chart.

Our research team has been working on the development of a computer-based, interactive, touchscreen self-management system designed for patient use in their homes. The system allows patients to assess, record, and track their vital signs, including weight, blood pressure, blood glucose level, temperature, and oxygen saturation (SpO_2_). The assessment records can be saved in the system and retrieved for review. The system can also remind the patients to take their prescribed medications at predetermined times. Figure 1 describes the measurement page for blood pressure. The page displays the blood pressure readings and includes the history data page button. By pressing the button, the users can access the history page and retrieve past blood pressure values from the two-dimensional line chart (see Figure 2). The design of the interface and functions of the other measurement modules (eg, blood glucose and weight) is similar to that of the blood pressure module. The intended users of the system are older adults with common chronic illnesses, such as diabetes, hypertension, and heart disease. The creation of the system interfaces was guided by a set of human factors design principles [4,39-41]. Examples of the principles are (1) match the system to the real world [4], (2) use recognition rather than recall [4], (3) reduce short-term memory load [39], (4) strive for consistency [39], (5) use compatibility of proximity principle [40], (6) conceptual compatibility [40], (7) avoid sound effects [41], (8) eliminate distracting features [41], and (9) have a clear and simple page [41]. In this study, we used a paper prototype that consisted of a collection of color-printed screenshots of the system interface to conduct our usability evaluation. This study protocol received the approval of the institutional review board of the University of Hong Kong. Informed consent was obtained from all of the participants.

### Heuristics Evaluation

Three important considerations were managed in our heuristics evaluation to ensure study quality: evaluators, heuristics, and evaluation process.

#### Evaluators

Our heuristics evaluation required the evaluators to be knowledgeable of usability and human factors engineering, be comfortable with health information system design and evaluation, be aware of the characteristics of older people (as the system end users would be elderly patients), and be familiar with the scenarios and environment in which the system would be used. In this study, we employed one “double expert” who had a background in usability and a domain of interest, and two “single experts” with experience in usability and human factors design. All of the evaluators were familiar with the heuristic principles. Nielsen and Mack [42] recommend using 3-5 “single experts” or 2-3 “double experts” in heuristics evaluations. We believed the number of evaluators in this study and their expertise level to be sufficient for this evaluation.

#### Heuristics

We evaluated our system interfaces for their conformity to a set of 26 human factors design heuristics (see Table 1) that were identified based on Nielsen [4], Shneiderman and Plaisant [39], Czaja and Lee [19], and Demiris and colleagues [41]. Because the heuristics of Nielsen and those of Shneiderman and Plaisant were general human-computer interface design heuristics, our evaluation also included the principles reported by Czaja and Lee and by Demiris and colleagues who developed heuristics specifically for older adults and elderly patients. The heuristics evaluation was conducted on December 15, 2011.

#### Evaluation Process

Three human factors researchers independently evaluated the conformity of the interface design to the 26 heuristics. They determined the conformity by responding “yes” or “no” to each heuristic. A comment section was also provided to collect their specific comments on the design issue associated with each heuristic. The three evaluators then met to discuss all of the comments received, identify the design problems, and give recommendations for system modifications prior to end-user testing.

### End-User Testing

The end-user testing was performed between January 16 and February 9, 2012, according to the three stages proposed by Nielsen [4], including preparation, testing, and follow up. Each test lasted approximately 30-40 minutes. The procedures implemented in these three stages are described below.

#### Preparation Stage

The preparation stage included participant selection, task design, and data collection.

##### Participants

The study participants were recruited from a non-profit medical organization in Hong Kong that provides medical services to the community in the Hong Kong East Cluster. The inclusion criteria for study participation included the following: (1) age 55 or older, (2) diagnosis of any chronic disease, (3) normal vision or corrected-to-normal vision, (4) no cognitive or physical impairment, and (5) the ability to read Traditional Chinese.

##### Task Design

The participants performed two practice tasks followed by 11 experimental tasks related to disease self-management (see Table 2). The tasks included a set of navigation tasks (tasks 1, 4, 5, 7, and 10) and a set of information search and simple cognitive tasks (tasks 2, 3, 6, 8, 9, and 11). In the navigation tasks, the participants were asked to access the measurement modules. To do this, they needed to search for and “press” the button associated with the module. In the information search and simple cognitive tasks, the participants were required to visually search for the measurement values (eg, blood glucose level) and to determine whether the values were normal based on the general “normal value range” presented on the interface.

##### Data Collection

Several performance measures were collected, including task incompletion rate, task completion time, frequency of error, and frequency of help. Task incompletion rate was defined as the percentage of participants who went through the task but were not able to complete it. Task completion time was the mean time it took to complete the task. The amount of time the participants had to complete the tasks was not limited, but they were instructed to try their best to perform the tasks. They were also asked to report to the research assistant (RA) if they were unable to complete the tasks. Frequency of error (n_error_) was defined as the total number of errors made on the task by all of the participants who went through the task (errors included choosing a wrong button, unable to find and interpret the information correctly, etc). The participants were corrected and were asked to try again when they made an error. Frequency of help (n_help_) was defined as the total number of times that all participants needed help on the task.

In addition, a questionnaire was administered in a face-to-face interview to examine the following variables: participant satisfaction with the system design (17 items), the perceived usefulness of the system (4 items), the perceived ease of use of the system (4 items), and the intention to use the system (1 item). The questionnaire was developed based on previous usability and technology acceptance studies [43,44]. Except for intention to use (which was a yes/no item with a follow-up question asking the participants to explain their responses), all other items were rated on 7-point Likert scales ranging from 1 = very bad to 7 = very good, 1 = strongly disagree to 7 = strongly agree, 1 = very unclear to 7 = very clear, 1 = very inappropriate to 7 = very appropriate, or 1 = very difficult to 7 = very easy. At the end of the interview, two open-ended questions were also asked to elicit the opinions of the participants about the interface design (eg, use of font size, color, and complexity) and about what they liked or did not like with the design.

#### Testing Stage

End-user testing was conducted in a community health service center by two trained RAs. Prior to the start of testing, one RA explained the study objective and research protocol to the participants. After the participants gave informed consent, the RA provided detailed information about the test procedures, described the purpose of the computer-based self-management system, and collected their basic demographical information. During the test, the participants were given two practice tasks to become familiar with the self-management system and the think-aloud method. Following the practice trials, the participants were asked to perform the experimental tasks. They were told to vocalize whatever they saw, did, and felt when performing the tasks. The participants did not go through the information search and simple cognitive tasks if they failed to complete the associated preceding navigation tasks. In this study, all end-user testing was recorded on video. The RAs also took field notes about the participants’ performance and comments. The RAs collected the questionnaire data and participant feedback on the difficulties they noticed when using the system after the completion of the end-user testing.

#### Follow-up Stage

In the follow-up stage, the study data were analyzed by two RAs using descriptive statistics and simple content analysis [45]. Data from the performance measures were extracted from the videos, and the means/frequencies were examined. Central tendency and distribution of the questionnaire item scores were determined. Audio interview data were transcribed and the content was analyzed. Practice task data were excluded from the data analysis.

**Table 1:** The 26 human factors design heuristics used in the heuristics evaluation.

Source	Heuristic
Nielsen [4]	1. Use simple and natural dialogue
	2. Speak the users’ language
	3. Provide clearly marked exits
	4. Provide help and documentation
Shneiderman and Plaisant [39]	5. Strive for consistency (eg, screen information location and operating procedures)
	6. Enable frequent users to use shortcuts
	7. Offer informative feedback
	8. Design dialogues to yield closure
	9. Offer simple error handling
	10. Permit easy reversal of actions
	11. Support internal locus of control
	12. Reduce short-term memory load
Czaja and Lee [19]	13. Maximize the contrast between characters and screen background
	14. Avoid small targets and characters that are small (fonts < 12 point)
	15. Minimize irrelevant screen information
	16. Adhere to principles of perceptual organization (eg, grouping)
	17. Highlight important screen information
	18. Clearly label keys
	19. Avoid color discriminations among colors of the same hue or in the blue-green range
	20. Maximize size of icons
	21. Use icons that are easily discriminated and meaningful, and label icons if possible
	22. Minimize demands on spatial memory
Demiris and colleagues [41]	23. Use proper visual display (eg, concrete symbols that should look like the object they represent and be distinguishable from others; large buttons that increase the area that can be selected with the pointer)
	24. Avoid sound effects
	25. Eliminate distracting features
	26. Use a simple and clear page

**Table 2:** Self-management tasks used during end-user testing.

Task #	Task description
Practice	Access the SpO_2 _measurement module
Practice	Indicate the SpO_2 _value and determine whether it is normal
1	Access the blood pressure measurement module
2	Indicate the systolic pressure value and determine whether it is normal
3	Indicate the diastolic pressure value and determine whether it is normal
4	Access the blood glucose measurement module
5	Select the “before breakfast” test time for blood glucose measurement^a^
6	Indicate the blood glucose value and determine whether it is normal
7	Access the body weight measurement module
8	Indicate the weight value
9	Indicate the body mass index (BMI) and determine whether it is normal
10	Access the history data page for blood pressure
11	Indicate the diastolic pressure value on a specified date on the history data chart

^a ^In this task, the participants had to search for and select (by “pressing”) the “before breakfast” button for the measurement.

## Results

### Heuristics Evaluation

The evaluation results (Table 3) and comments of the three evaluators were discussed and compiled into four categories (Table 4). The evaluation identified some strengths in the system design, such as consistent information presentation and organization, low demand on user short-term and spatial memory load, clearly labeled keys, and consistent operating procedures within and across the system modules. Two types of usability problems were also identified. The first was general usability problems related to insufficient interface design, including unfamiliar terminology, confusing and inconsistent button design, lack of informative feedback for user actions, and a lack of online support and instruction. The second type was age-related usability problems that were more problematic for older adult patients due in part to small text and buttons, inappropriate use of serif font and gradient color, low contrast between information and background, and too much information on the interface. Based on these findings, changes were made to the system design for end-user testing.

### End-User Testing

#### Participant Characteristics

A total of 57 eligible older adult patients participated. The first seven were pilot participants to try out the testing procedures through which the experimental design problems were identified and fixed prior to the main test. The other 50 participants completed the main test; only their data were used for analysis. Table 5 presents the characteristics of the participants.

#### Performance Measures

The performance data were analyzed with descriptive statistics. Table 6 shows the results.

**Table 3:** Heuristics evaluation results.^a^

Heuristic	Interface^b^									
	a	b	c	d	e	f	g	h	i	j
1	✓	✓	✓	•	✓	✓	✓	✓	✓	✓
2	✓	✓	✓	✓	✓	✓	✓	✓	•	✓
3	NA	✓	✓	✓	✓	✓	✓	✓	✓	✓
4	✗	✗	✗	✗	✗	✗	✗	✗	✗	✗
5	✓	•	•	•	•	•	•	•	•	•
6	NA	NA	NA	NA	NA	NA	NA	NA	NA	NA
7	✓	•	✓	✓	•	✓	•	✓	•	✓
8	NA	NA	NA	NA	NA	NA	NA	NA	NA	NA
9	✓	✓	✓	✓	✓	✓	✓	✓	✓	✓
10	✓	✓	✓	•	✓	•	✓	•	✓	•
11	✓	✓	✓	✓	✓	✓	✓	✓	✓	✓
12	✓	✓	•	✓	✓	✓	✓	✓	✓	✓
13	✓	•	✓	✓	•	✓	•	✓	•	✓
14	✓	✗	✗	✗	✗	✗	✗	✗	✗	✗
15	✓	✗	✗	✗	✗	✗	✗	✗	✗	✗
16	✓	✓	✓	✓	✓	✓	✓	✓	✓	✓
17	✓	✓	✓	✓	✓	✓	✓	✓	✓	✓
18	✓	✓	✓	✓	✓	✓	✓	✓	✓	✓
19	✓	•	✓	✓	•	✓	•	✓	•	✓
20	✓	•	•	•	•	•	•	•	•	•
21	•	•	✓	•	•	✓	•	✓	•	•
22	✓	✓	•	✓	✓	✓	✓	✓	✓	✓
23	✗	✗	✗	✗	✗	✗	✗	✗	✗	✗
24	✓	✓	✓	✓	✓	✓	✓	✓	✓	✓
25	✓	✗	✗	✗	✗	✗	✗	✗	✗	✗
26	✓	✗	✗	✗	✗	✗	✗	✗	✗	✗

^a ^✓: All three evaluators verified the conformity; •: only one or two of the evaluators verified the conformity, but the other evaluator(s) expressed nonconformity; ✗: none of the evaluators verified the conformity; NA: the heuristic was not applicable to the design of the interface.

^b ^a: System home page with six measurement module buttons; b: blood pressure measurement page; c: history data page for blood pressure; d: blood glucose test time selection page; e: blood glucose measurement page; f: history data page for blood glucose; g: SpO_2 _measurement page; h: history data page for SpO_2_; i: body weight measurement page; j: history data page for body weight and BMI.

**Table 4:** Interface design strengths and usability problems identified in the heuristics evaluation.

Category	Strengths	General usability problems	Age-related usability problems
Readability	High contrast between most characters and background	Unfamiliar terminology	Small characters, texts, and buttons
		Confusing design of the navigation buttons and their icons	Low contrast between some numbers and background
			Inappropriate use of serif fonts and gradient colors
Information presentation	Consistent information presentation and organization	Unclear reference information and icons	Irrelevant screen information and too much information on one interface
	Adherence to principles of perceptual organization when grouping information	Inappropriate layout of some interface elements	Inappropriate use of green color to display information
	High conspicuity of important information		
Information retrieval and interpretation	Low demand on the user’s spatial memory	Lack of informative feedback for users’ actions	Lack of hints for older adults to find information
	Clearly labeled keys	Lack of online support and instruction on how to use the system	
	Consistent operating procedures with and across the system modules	No error message	
	No complex command language		
Navigation		Inconsistent button design	

**Table 5:** Study participant characteristics (N = 50).

Characteristics		n (%) or mean (SD)	
**Gender, n (%)**		
	Male	15 (30%)
	Female	35 (70%)
Mean age (SD)		71.6 (9.7)
**Previous technology experience, n (%)**		
	Experience using personal computers	17 (34%)
	Experience using a touch screen computer	6 (12%)
Average weekly personal computer use, hours (SD)		4.1 (6.0)
Experience using any computer-based disease self-management system (n)		0
**Chronic diseases diagnosed, n (%)** **^a^**		
	Hypertension	40 (80%)
	Diabetes	22 (44%)
	Heart disease	11 (22%)
	Asthma	3 (6%)
	Prostatitis	2 (4%)
	Hypotension	2 (4%)

^a ^Twenty-seven (54%) of the participants reported having two or more of the chronic diseases.

**Table 6:** Performance measures as assessed via 11 tasks.

Task		n	Task incompletion rate	Mean task completion time (sec)	Frequency of error n_error _(n)^a^	Frequency of help n_help _(n)^b^
**Navigation tasks**						
	1	50	0%	12.6	4 (4)	22 (15)
	4	50	0%	14.1	6 (6)	18 (11)
	5	50	16%	23.0	39 (19)	48 (24)
	7	50	2%	8.5	5 (5)	12 (7)
	10	50	44%	58.4	93 (45)	60 (28)
**Information search and simple cognitive tasks**						
	2	50	28%	6.7	18 (14)	18 (13)
	3	50	34%	5.2	22 (16)	16 (6)
	6	42	26%	7.2	20 (16)	13 (5)
	8	49	22%	10.2	16 (12)	18 (8)
	9	48	17%	12.2	14 (10)	26 (16)
	11	28	50%	10.5	28 (17)	18 (12)

^a ^n_error _represents the number of times an error was made and n represents the number of people who made the error.

^b ^n_help _represents the number of times help was given and n represents the number of people who needed help.

##### Task Incompletion Rate

All participants completed all of the navigation tasks due to the nature of our experimental design; however, not everyone completed all of the information search and simple cognitive tasks because they failed to complete the preceding navigation tasks. For instance, only 42 participants completed task 6 because 8 participants failed to complete task 5. In the navigation tasks, tasks 1 and 4 yielded a task incompletion rate of 0%. A low incompletion rate (2%, 1/50) was yielded in task 7. However, task 10 had an incompletion rate of 44% (22/50). Task incompletion rates were moderate to high for the information search and simple cognitive tasks, ranging from 17% (8/48) to 50% (14/28), respectively. For example, half of the 28 participants were unable to complete task 11 (50% incompletion rate).

##### Task Completion Time

Among all of the navigation tasks, tasks 1, 4, and 7, which required the participants to access the measurement modules, yielded the shortest task completion times. The “access the history data page” task and “select the breakfast test time” task appeared to be difficult to perform, with fairly long task completion times. Among the 11 experimental tasks, tasks 2, 3, and 6, which required the participants to indicate a vital sign value and determine whether it was normal, had the shortest completion times. Task 9 (indicate the BMI and determine its normality), task 11 (read the history data chart and find the diastolic pressure value), and task 8 (indicate the weight value) yielded longer completion times.

##### Frequency of Error

Both navigation errors (eg, choosing wrong navigation buttons, incorrectly recognizing icons and symbols as buttons, and failing to follow the navigation paths) and information processing errors (eg, failing to locate and explain information; being unable to retrieve the measurement values, such as the blood glucose value; and being unable to obtain and comprehend the reference values of normal blood pressure levels) were observed. Overall, 93 errors (highest occurrence among all tasks) were made by 45/50 participants in the “access the history data page” task. The task that required the participants to select (by “pressing”) the “before breakfast” test time for measuring their blood glucose levels yielded the second highest number of errors (39 errors made by 19/50 participants). The information search and simple cognitive tasks yielded a moderate frequency of errors.

##### Frequency of Help

Similar to the frequency of error finding, tasks 5 and 10 yielded the highest frequency of help, indicating that the tasks were difficult based on our current design. For instance, 28 participants (56%) needed help a total of 60 times when doing the “access the history data page” task.

#### Satisfaction, Perceived Usefulness, and Perceived Ease of Use

Table 7 presents the central tendency and distribution of the questionnaire responses. The mean scores for satisfaction, perceived usefulness, and perceived ease of use were at least 4.9 (SD 1.4), 6.0 (SD 1.2), and 6.0 (SD 1.2), respectively. All of these were above the midpoint of the scale, indicating that the participant exhibited a positive impression of the system design. Of the 17 satisfaction items, 14 had a mean score of 6.0 or higher. The mean ratings of two satisfaction items (Sat2: the information on the interfaces are overloaded, and Sat9: finding information on this system requires a lot of mental effort) were relatively low, showing that the amount of information on the interface might be excessive and that finding this information required a large amount of mental effort.

**Table 7:** Descriptive statistics for satisfaction, perceived usefulness, and ease of use items (1 = negative to 7 = positive).

Item		Rating distribution, n (%)							Mean	SD
		1	2	3	4	5	6	7		
**Satisfaction**										
	Sat1: System appearance	0 (0%)	1 (2%)	1 (2%)	4 (8%)	5 (10%)	23 (46%)	16 (32%)	5.9	1.1
	Sat2: Amount of information	0 (0%)	3 (6%)	3 (6%)	18 (36%)	10 (20%)	7 (14%)	9 (18%)	4.9	1.4
	Sat3: Graphic quality	0 (0%)	2 (4%)	0 (0%)	1 (2%)	3 (6%)	19 (38%)	25 (50%)	6.2	1.1
	Sat4: Character size	0 (0%)	0 (0%)	1 (2%)	0 (0%)	5 (10%)	19 (38%)	25 (50%)	6.3	0.8
	Sat5: Ease of reading the information	1 (2%)	0 (0%)	2 (4%)	2 (4%)	4 (8%)	18 (36%)	23 (46%)	6.1	1.3
	Sat6: Text clarity/understanding	0 (0%)	0 (0%)	2 (4%)	1 (2%)	1 (2%)	13 (26%)	33 (66%)	6.5	1.0
	Sat7: Congruence between information and expectations	0 (0%)	0 (0%)	1 (2%)	2 (4%)	2 (4%)	21 (42%)	24 (48%)	6.3	0.9
	Sat8: Ease of finding information	0 (0%)	1 (2%)	0 (0%)	3 (6%)	3 (6%)	14 (28%)	29 (58%)	6.4	1.0
	Sat9: Mental efforts in finding information	1 (2%)	0 (0%)	4 (8%)	4 (8%)	19 (38%)	11 (22%)	11 (22%)	5.3	1.3
	Sat10: Helpful for finding health information	0 (0%)	1 (2%)	0 (0%)	1 (2%)	4 (8%)	12 (24%)	32 (64%)	6.4	1.0
	Sat11: Helpful for understanding health problems	1 (2%)	2 (4%)	2 (4%)	3 (6%)	5 (10%)	12 (24%)	25 (50%)	6.0	1.5
	Sat12: Improvement in health knowledge	0 (0%)	1 (2%)	1 (2%)	1 (2%)	3 (6%)	12 (24%)	32 (64%)	6.4	1.1
	Sat13: Improvement in knowledge of chronic illness and their treatment	1 (2%)	0 (0%)	2 (4%)	1 (2%)	5 (10%)	9 (18%)	32 (64%)	6.3	1.3
	Sat14: Easier and more efficient at self-management	0 (0%)	0 (0%)	2 (4%)	3 (6%)	3 (6%)	12 (24%)	30 (60%)	6.3	1.1
	Sat15: Encouragement to taking better care	0 (0%)	1 (2%)	1 (2%)	4 (8%)	3 (6%)	14 (28%)	27 (54%)	6.2	1.2
	Sat16: Helpful for performing better self-care	0 (0%)	1 (2%)	1 (2%)	1 (2%)	9 (18%)	13 (26%)	25 (50%)	6.2	1.1
	Sat17: Saving time in self-management	0 (0%)	1 (2%)	2 (4%)	1 (2%)	7 (14%)	9 (18%)	30 (60%)	6.3	1.1
**Perceived usefulness**										
	U1: Improvement of ability to self-management	0 (0%)	1 (2%)	0 (0%)	3 (6%)	9 (18%)	15 (30%)	22 (44%)	6.1	0.9
	U2: Time saving in self-management	0 (0%)	1 (2%)	1 (2%)	6 (12%)	8 (16%)	10 (20%)	24 (48%)	6.0	1.2
	U3: Effectiveness of self-management	0 (0%)	1 (2%)	3 (6%)	3 (6%)	4 (8%)	16 (32%)	23 (46%)	6.1	1.2
	U4: Usefulness for self-management	0 (0%)	0 (0%)	0 (0%)	3 (6%)	7 (14%)	15 (30%)	25 (50%)	6.3	0.9
**Perceived ease of use**										
	EOU1: Ease of learning the system	0 (0%)	0 (0%)	0 (0%)	2 (4%)	4 (8%)	19 (38%)	25 (50%)	6.3	0.8
	EOU2: Ease of getting the system to do tasks	0 (0%)	1 (2%)	2 (4%)	1 (2%)	9 (18%)	17 (34%)	20 (40%)	6.0	1.2
	EOU3: Ease of being skillful at using the system	0 (0%)	0 (0%)	1 (2%)	4 (8%)	5 (10%)	20 (40%)	20 (40%)	6.1	1.0
	EOU4: Ease of using the system	0 (0%)	0 (0%)	1 (2%)	1 (2%)	6 (12%)	22 (44%)	20 (40%)	6.2	0.9

#### Intention to Use the System

Thirty-one (74%) participants expressed their intention to use the actual system for chronic disease self-management in the future, if the system was available. The reasons listed for wanting to use the system were that the system could facilitate their self-management of chronic diseases, such as providing them with specific and updated health information; automatically recording the health information for easy retrieval later saving time on their self-management; and improving communication with their health care providers. For those who said that they would not use the system, cost, unfamiliarity with the technology, and limited space at home for the system were the major reasons cited for non-use.

#### Comments from Open-Ended Questions

All participants expressed a fondness for the system. They commented that the overall system interface was effective and appealing, the system was simple to use, the information on the interfaces was clearly presented, and using the system for self-management would allow them to obtain useful health information and improve their health conditions. However, comments related to usability problems were also mentioned. They were grouped into four categories and are presented in Table 8. Although some of the problems were similar to those identified in the heuristics evaluation (eg, unfamiliar terminology, small characters and texts, and inconsistent button design), the comments offered more details about the design that enabled us to develop specific directions for system redesign.

**Table 8:** Usability problems identified from the open-ended questions.

Category	Usability problem (n)^a^
Readability	Characters too small and words too busy (3)
	Low-quality graphics (10)
	Too small icons and words that were placed over the buttons (3)
	Low contrast of the color indicators (4)
	Too small decimal point symbol of the numbers (4)
	Inappropriate use of color in color indicators (7)
Information presentation	Inappropriate use of button icons (4)
	Complex design of the history data page interface (3)
	Unclear abbreviations and terminologies, such as “SpO_2_” (16), the unit “kg” (1), and “BMI” (1)
	Unnecessary icons on navigation buttons (6)
	Obscure reference information (5)
Information retrieval and interpretation	Ambiguous emoticons, which were used to facilitate participants’ information comprehension (14)
	Ambiguous information on “normal value range” presentation (14)
	Poor pairing design between the measurement value and its measurement date in the history data chart (9)
Navigation	Difficulty in choosing test time for blood glucose (11)
	Difficulty in accessing the history data page of blood pressure (27)
	Ambiguous design of the history page button because of its inconsistency with other buttons (10)
	Complex navigation between different measurement modules (2)

^a ^n = number of participants who expressed the problem.

## Discussion

This study assessed the interface design of a computer-based chronic disease self-management system using a set of design heuristics and evaluated the performance and perceptions of users about the system. Using the paper prototype, our evaluations quickly and effectively identified the system’s strengths and usability weaknesses.

### System Interface Design

Overall, our findings indicated that the participants were basically able to perform the study tasks using the current design. However, we also identified a number of design problems and areas that could be improved to further enhance usability. Moreover, based on our findings, we drew a number of long-reaching and significant implications on usability design guidelines for designing health IT systems for the elderly.

First, all four performance indicators showed that the “access the history data page” task (task 10) was difficult to perform. This was likely due to a design inconsistency where the appearance and position of the history page button was completely different from that of the six main measurement module buttons, as indicated by the findings of the heuristics evaluation and end-user test (ie, ambiguous design of the history page button). Because of this difference, when the participants performed the task, many of them attempted to find the button in the area where the six module buttons were grouped; therefore, the participants did not notice that the button was actually located in a different area of the interface. This inconsistency led to confusion and resulted in additional search efforts that would not be necessary if the location was changed. This finding confirms the design principle that the appearance, position, and configuration should be consistent across objects/displays (eg, buttons, icons) that serve the same basic functions (eg, going to a new page/module).

**Figure 3:**
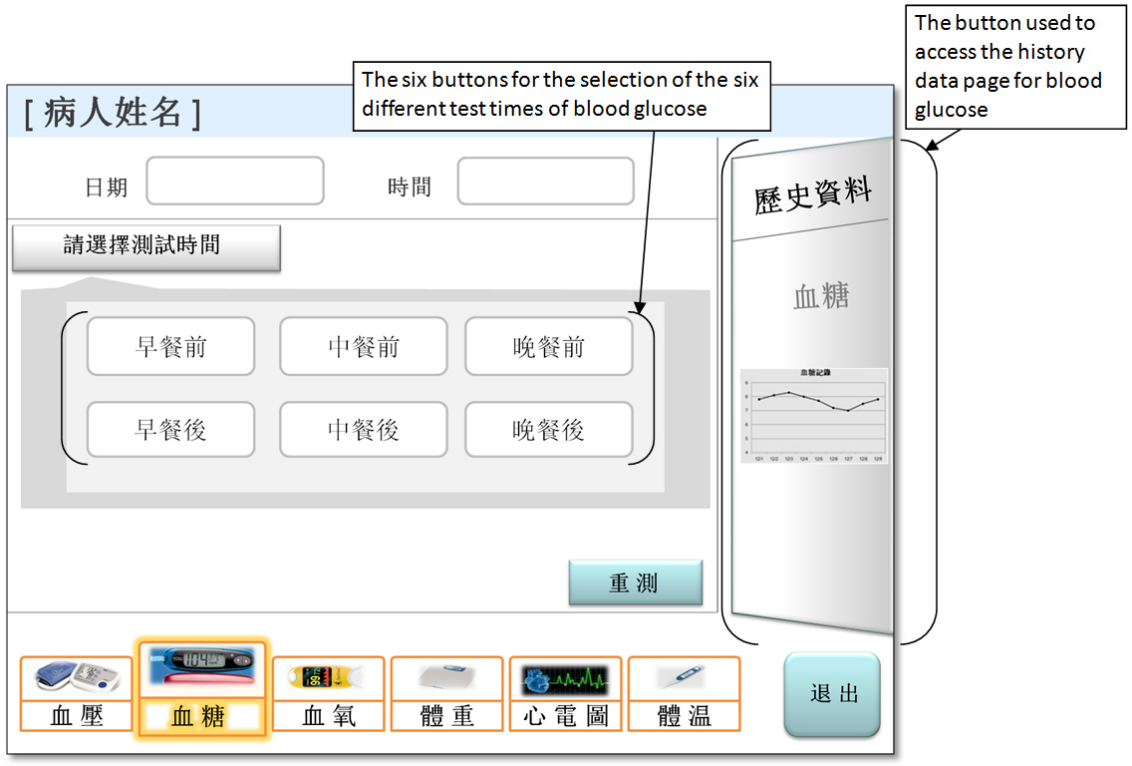
The blood glucose module menu includes the six buttons for the selection of the six test times of blood glucose.

Second, the blood glucose test time selection task (task 5) was also challenging because it had a similar inconsistent design problem. Furthermore, in the blood glucose module menu, there were a total of six alternative test times available for selection because the timing of the test could be before or after breakfast, lunch, or dinner (see Figure 3). Based on the participants’ comments about the end-user test and our observation, it appeared that the menu offered too many choices that added decision complexity (see the Hick-Hyman Law [46,47]). Additionally, older adults may experience declines in cognitive abilities and eyesight that can make it more difficult to process complex information and locate information on complex interfaces [19]. Our sophisticated menu, with its six options, likely required more visual search and cognitive effort for information processing and may have contributed to the lower task performance of the elderly patients. From this observation, we suggest that the number of choices in a menu/interface be kept to an essential minimum. Therefore, we modified our design such that the system would automatically record the test time.

Third, although the history data chart followed a simple two-dimensional line chart design in which the measurement dates were displayed along the x-axis and the measurement values were plotted along the y-axis, most elderly participants could not easily comprehend the chart and retrieve the values, as indicated by the performance data and the participants’ comments (ie, poor pairing design between the measurement value and its measurement date in the history data chart). This type of graphical representation can be especially difficult for older adults to read and comprehend. This finding suggests that when a graphical representation of measurement data is employed, it should be designed to help improve the older adults’ ability to pair the measurement dates with the corresponding measurement values.

Fourth, readability was another design weakness identified. The size of the fonts (all the Chinese characters) were set at 18 points in the original design. Although the literature recommended that font sizes be at least 14 points (eg, Demiris and colleauges [41]), the findings of the end-user test showed that when Chinese characters were used, an 18-point font size was too small for the older adults to read due to the crowded strokes in the characters. Moreover, the sizes of the icons and symbols were too small. These findings suggest that the fonts, icons, and symbols should be larger for the elderly population. While the mean score of the satisfaction item that examined the graphic quality (Sat3: overall quality of graphics) was high, the participants’ comments about the end-user test indicated that the picture quality of the icons and symbols was inadequate and that affected the overall readability. Therefore, high image resolution should be used in icons and symbols.

Fifth, regarding the presentation of information, a number of participants expressed their confusion about the pictures that were used to describe the functions of the buttons (eg, a picture of a scale was used to represent weight measurement). The selection and use of these icons should be revisited and meaningful pictures should be used to enhance the conceptual compatibility. Additionally, both the heuristics evaluation and end users’ comments indicated that the abbreviations and some of the medical terminologies used in the interfaces (eg, SpO_2 _and BMI) were unclear and too technical. The older adults in particular may not have the knowledge to understand the meanings of these terms. Therefore, they should be replaced with plain, non-technical terms that are less ambiguous to users.

### Usability Test Methodology and Design

A number of research methodology and design issues are worth discussing because these provide important implications for health information technology usability research. First, although using computer-based interactive system prototypes in the usability test can allow researchers to measure realistic user interactions, evidence shows that paper prototypes are as effective as computer-based prototypes in uncovering usability problems and understanding the users’ subjective evaluation of a system [36,48,49]. Moreover, paper prototypes are less costly and can be created quickly.

Second, many of the previous studies adopted a single usability testing method. Our findings revealed a number of usability issues, not detected in the heuristics evaluation, discovered by the end-user testing. Furthermore, our heuristics evaluation only projected high-level structural usability problems (eg, font size and information grouping problems), whereas the end-user testing allowed us to discover a large number of usability weaknesses at detailed levels. Our study showed that using multiple evaluation approaches could help identify more potential problems and should be a more reliable practice for conducting usability studies (also noted in the literature; see [30,33]).

Third, one of the main criticisms of previous studies on health IT usability has been the lack of a theoretical basis for the development of the study methodology [50]. Our study method and procedures were carefully set up based on systematic usability study guidelines and models as well as empirical research, such as Nielsen and Mack [42] and Nielsen [4]. These guidelines provided valid directions for our experimental design and prevented erroneous testing protocols and data collection.

Fourth, effective disease self-management systems have the potential to improve care quality and safety [51,52]. However, one cannot meaningfully examine and then be certain of the true value of a newly developed health information system (such as the one in this current study) without having the usability and design problems discovered and eliminated beforehand. For instance, a system with an unpleasant and ineffective interface design found to have no impact on health outcomes could actually be beneficial if the design weaknesses had been overcome prior to the examination. Our study suggests that the usability test is one of the steps that should be performed during the system development process to avoid drawing mistaken conclusions about system effectiveness.

### Strengths and Limitations

Our study had several strengths: (1) careful and systematic procedures were adopted in the heuristics evaluation and end-user testing; (2) context-specific consideration was exercised to generate heuristics for the heuristics evaluation and to develop performance measures in the end-user testing; and (3) compared to many other usability studies, our study involved a relatively large sample size, which may have helped identify more usability problems and design weaknesses of our system. However, a few limitations should be noted. First, no alternative system interfaces were assessed; therefore, no comparable results were available on a better interface design approach in our study. This may limit our findings and ideas on system interface improvement. Second, although it was not the focus of this current study, it may be worth considering the effects of user characteristics (eg, age, severity of the chronic conditions, and computer experience) on measurement outcomes. Third, it may be valuable to verify the effectiveness of our design recommendations by conducting iterative usability evaluations. However, we are planning to conduct another round of usability studies using a computerized prototype with the design recommendations incorporated.

### Conclusions

An inadequately designed health information system increases the likelihood of the users committing user errors and having difficulties using the system. These issues can be mitigated by identifying a system’s usability problems using heuristics evaluations and end-user tests, and the results of these evaluations can be used for design refinement. Importantly, special attention should be given to the selection of design heuristics for evaluating systems for elderly patients because the general human factors design guidelines may be insufficient for addressing the unique characteristics and capabilities of elderly patients. Furthermore, the design problems discovered in this study allow for the implementation of new design guidelines that are of particular importance for the elderly and can be generalized to other health information systems that are designed for older adult patients.

## Abbreviations

BMI: body mass index
IT: information technology
RA: research assistant
SpO2: oxygen saturation
